# Identifying Health Care Services Offered in the HIV Care Continuum via a Machine Learning–Based Topic Modeling Approach: Exploratory Literature Review

**DOI:** 10.2196/65081

**Published:** 2025-07-09

**Authors:** SangA Lee, Layoung Kim, Mi-So Shim, Gwang Suk Kim

**Affiliations:** 1 Mo-Im Kim Nursing Research Institute, College of Nursing, Yonsei University, 50-1 Yonsei-ro, Seodaemun-gu, Seoul, 03722, Republic of Korea, 82 2-2228-3342, 82 2-2227-8303; 2 College of Nursing and Brain Korea 21 FOUR Project, Yonsei University, Seoul, Republic of Korea; 3 College of Nursing, Keimyung University, Daegu, Republic of Korea

**Keywords:** HIV infections, continuity of patient care, health services, review, machine learning

## Abstract

**Background:**

It remains unclear whether the existing health care services reflect the HIV care continuum, which underscores the need for integrated care beyond viral suppression.

**Objective:**

This study aimed to analyze the literature on health care services for people living with HIV to enhance the understanding of trends and knowledge structures.

**Methods:**

A literature review was conducted using BERTopic, an advanced machine learning-based topic modeling technique. We searched PubMed, CINAHL, EMBASE, and Cochrane databases for English-language studies published between 2013 and 2023. Analyses were performed twice: first, to gain a broad understanding of the literature, and second, to examine the specific details of health care services described.

**Results:**

Among the 11,269 articles screened, 204 studies met the inclusion criteria. Within the HIV care continuum, most studies focused on the treatment retention stage, while studies focusing on the long-term stage were limited. A broad literature analysis identified five key topics, with “ART adherence” emerging as the most prominent topic. A more comprehensive analysis of health care services within the literature revealed 7 topics, reflecting diverse delivery methods and content in providing health care services for people living with HIV. The predominant topic, “ART adherence and counseling,” encompassed the largest number of studies, indicating the strongest emphasis in the field. Notably, the distribution of topics exhibited a distinct pattern: while health care service diversity was the highest in the earlier stages of the HIV care continuum, it became increasingly limited in the later stages.

**Conclusions:**

This study provides valuable insights into current HIV care services and highlights areas for future research and intervention. Despite the shift toward lifelong HIV management, existing literature remains heavily focused on medication treatment, overlooking the multifaceted health care needs of people living with HIV. Research disparities, particularly concerning vulnerable populations, underscore the need for more inclusive studies and tailored health care services. Efforts should be intensified to bridge these gaps, ensuring inclusive and equitable health care services across diverse populations and fostering interdisciplinary collaboration to meet the evolving needs of people living with HIV, thereby enhancing the HIV care continuum for all.

## Introduction

Similar to other chronic illnesses, people living with HIV require a lifelong disease-specific regimen to maintain their physical, psychological, and social well-being [1]. While individuals with sustained viral suppression do not risk sexually transmitting the virus to others [2], those with poor psychological health and lower socioeconomic status—measured by factors such as income, housing, and education—are particularly vulnerable to dropping out of care. This vulnerability is exacerbated by HIV-specific barriers, including stigma, discrimination in health care settings, and individual risk factors [3-5]. To address these challenges, health care services must be scaled up to provide targeted support [3,4]. Moreover, as these barriers cannot be immediately eliminated, a more continuous and comprehensive approach to HIV treatment and life journey is necessary.

The global response to HIV treatment has focused on retaining people living with HIV in care to ensure their healthy lives and to end AIDS [6]. In 2013, the HIV care continuum was established to outline the steps for the continuity of treatment outcomes, namely: diagnosis, linkage to care, treatment initiation, treatment retention, and the achievement and maintenance of viral suppression [7,8]. Institutions such as the World Health Organization (WHO) [9], International Association of Providers of AIDS Care [10], and US Department of Health and Human Services [11] proposed various guidelines, including health care services for people living with HIV on the care continuum at the international and national levels. These guidelines have agreed on the importance of the HIV care continuum, which requires a broader scope of integrated HIV care to achieve holistic health beyond viral suppression [12].

In the context of continuity, the WHO highlights that integrated, people-centered care expands the capacity of patients to play a more active role in long-term survival [13]. Positioned as components of the HIV care continuum, health care services such as HIV testing, medication supply, antiretroviral therapy (ART) adherence monitoring, adverse event management, immunological and virological monitoring, and follow-up for missed visits have been explored in previous studies [14-16]. However, it remains unclear whether the services being offered to people living with HIV align with lifelong continuity of care. To provide effective health care services supporting the HIV care continuum, a comprehensive data review to gain an in-depth understanding of historical trends and key perspectives on the services being provided to people living with HIV must be conducted. There are global, regional, and national differences in coverage, HIV incidence, prevalence, and mortality [17]. Low-income countries have focused on high-priority services, such as child and maternal health, due to resource constraints and immediate health care needs; whereas high-income countries possess the infrastructure, funding, and policy frameworks necessary to support more advanced health care systems. In particular, high-income countries have prioritized the development of integrated care, a prominent policy theme aimed at addressing complex and interconnected health issues, including chronic diseases and the HIV care continuum [18]. Furthermore, high-income countries often serve as benchmarks for global policy development, making their trends and outcomes particularly valuable for informing evaluation and service provision [19]. Their well-established systems, combined with consistent access to resources and comprehensive data, enable the study of integrated care systems in a way that minimizes variability and reduces potential confounding factors associated with differences in economic conditions or institutional capacities. This focus on high-income countries ensures greater comparability and facilitates the derivation of meaningful, context-specific insights. Therefore, health care services provided in high-income countries [20] need to be analyzed first to review the HIV care continuum from the long-term perspective.

As various HIV health care service studies are being conducted across treatment stages, individuals, and methods, the literature needs to be comprehensively reviewed. Traditional methods, such as manual literature reviews and qualitative content analysis, while valuable for deriving specific insights from research criteria, face significant limitations in handling large and complex datasets. These methods are time-intensive, rely heavily on human expertise, and are subjective to judgment, making them prone to inconsistency as well as bias [21,22]. Furthermore, these methods are less effective at detecting overarching trends, correlations, and topic evolution over time, particularly in diverse and extensive datasets. Manual keyword extraction and topic grouping often fail to fully capture nuanced relationships between concepts, limiting the ability to generate a comprehensive and graphical perspective of the literature. To review, summarize, classify, and detect trends more efficiently in large-scale documents, text analysis techniques offer a valuable and effective solution [23,24].

To address these challenges, this study used Bidirectional Encoder Representations from Transformers (BERT) developed by Devlin [25] in Google artificial intelligence (AI) Language. Among various text analysis technical models, BERT is recognized as one of the most recent and advanced machine learning-based approaches to topic modeling. Unlike traditional text analysis methods, techniques like BERT offer several advantages. Its ability to pretrain deep bidirectional representations enables BERT to capture the contextual meanings of words and sentences by considering both left and right contexts simultaneously [25], resulting in more accurate and nuanced topic modeling. An example of traditional methods is the Latent Dirichlet Allocation (LDA), which relies on simpler probabilistic frameworks and often fails to account for complex semantic relationships [25,26]. Studies comparing LDA and BERT have demonstrated that BERT outperforms LDA in extracting deeper and distinct topics, identifying intricate topic relationships in datasets with complex linguistic structures [27,28]. BERT is particularly effective at processing large datasets, efficiently summarizing, classifying, and detecting trends across a wide range of documents. Considering its strengths, it has been increasingly used in research, particularly literature reviews analyzing numerous studies as well as social media analyses processing numerous tweets to extract insights on health care issues [29-32]. Its scalability reduces the time and resources required for manual analysis while ensuring consistency in topic extraction and classification. In addition, BERT excels in modeling topic correlations as well as their evolution over time, providing a dynamic and graphical perspective of the data that traditional methods struggle to achieve. Its ability to handle complex linguistic structures and low-resource tasks enables the identification of meaningful patterns in diverse contexts, further enhancing its utility for analyzing comprehensive datasets [33]. While BERT offers numerous strengths, it effectively addresses the limitations of traditional approaches by ensuring objectivity, scalability, and the ability to generate insights that would otherwise require extensive human effort and expertise. These capabilities make BERT particularly well-suited for reviewing the extensive and heterogeneous body of literature on HIV health care services. This study therefore used BERT to depict the bibliographic trends and knowledge structure of publications on health care services to improve the HIV care continuum for people living with HIV in high-income countries.

## Methods

### Design

This study used a quantitative content analysis using BERTopic modeling to examine the keywords and topics in the literature on health care services provided to people living with HIV. The methodological process comprised phases: (1) search strategy and selection, (2) data preprocessing, and (3) data analysis, using topic modeling with BERTopic.

### Search Strategy and Selection Process

Relevant literature was searched from PubMed, CINAHL, EMBASE, and Cochrane databases. Key search terms included Medical Subject Heading (MeSH) terms, trade words, and natural language words such as “care continuum,” “HIV/AIDS,” and “service.” The search was limited to studies published between January 2013—when the WHO first introduced comprehensive guidelines on ART for all populations across the HIV care continuum—and December 2023 [34]. The inclusion criteria were studies: (1) targeting adults diagnosed with HIV, (2) investigating or providing health care services (eg, interventions, services, or programs) to improve the HIV care continuum, and (3) any study design conducted in high-income countries. The exclusion criteria were: (1) studies targeting specific subpopulations (eg, drug users, individuals with mental illnesses, or sex workers), (2) studies targeting HIV prevention services, (3) studies for which the full text was unavailable in English, (4) studies targeting public policies, (5) literature reviews, and (6) duplicates. The exclusion of studies on specific subpopulations aimed to focus the review on general HIV care rather than focusing on tailored interventions for specific groups. While these populations face unique challenges and require specialized interventions, their exclusion was conducted to provide a broader perspective on the HIV care continuum. This approach ensures that the analysis reflects the overall capacities and integration efforts of health care systems, offering insights applicable to the wider population rather than a subset of individuals.

In total, 14,377 articles were retrieved from the databases. After removing duplicates, 11,269 articles were screened for their titles, abstracts, and full texts to determine their eligibility. Multiple studies using the same health care service were assessed through text frequency analysis to detect keyword matches. Most exhibited few keyword matches, suggesting distinct studies. Subsequently, all studies, with the exception of secondary analyses, underwent a full-text review. Three researchers (SL, MS, and LK) independently screened all the articles and selected the final list of articles for analysis after discussion. Discrepancies were discussed with the principal investigator (GK) and consensus was reached as to whether to include the article. The dataset size selected was deemed appropriate for BERT, as it demonstrated efficacy in handling modest-sized datasets with a comparable scale in previous studies, often using 100 documents [28,35]. As with many bibliometric analysis studies exploring research trends [36], a quality assessment was unnecessary. All the data, including the metadata elements of the title, abstract, authors, and year, were downloaded for analysis.

### Data Preprocessing

Before data analysis, a sequence of preprocessing steps were conducted to clean and prepare the data for further analysis. Articles without abstracts were excluded to ensure a comprehensive set of textual information. Additional columns were created to classify studies by country, HIV care continuum, and health care services provided. The countries from which each study collected data and the specific stages of the HIV care continuum on which each study focused were identified to derive more meaningful interpretations of the distribution of research topics. Specific stages of the HIV care continuum, including linkage to care, treatment initiation, treatment retention, and the long-term stage [7,8], were identified. The classification was finalized following a consensus reached through discussion between 2 researchers (SL and LK), with multiple stages being investigated in one study. After incorporating studies covering multiple stages, the final corpus comprised 242 documents for analysis. To ensure a more thorough investigation of health care services, detailed descriptions of services from the methods sections of each article were extracted. This process, conducted by 2 researchers (SL and LK), enhanced the accuracy of the dataset. The usage of details regarding the health care services described in the methods section helped create a more robust text corpus, enhancing the efficacy of the subsequent topic modeling analysis.

Stop words were then excluded, and a replacement dictionary was generated. Stop words, including “a,” “the,” and “of,” refer to common words that appear frequently in the text but do not convey insights into the specific topic of a document. All English stop words were removed using the Natural Language Toolkit library in Python. Words that barely contained significant meanings for the research purpose and degraded or hindered the interpretability of results (eg, background, method, results, and trial registration) were added to the toolkit. The extended stop words were then excluded from the data. The replacement dictionary was used to combine several words or abbreviations with the same or similar meanings and designate a single representative word (eg, people living with HIV, HIV, and AIDS). During this stage, repeated word analyses were performed with regard to abstract, and the researchers reached consensus on the words to be registered in the dictionaries through discussion.

### Data Analysis

BERTopic, a Python-based package, was used to extract the primary topics from the literature. It was implemented with the all-MiniLM-L6-v2 embedding model to ensure accurate and efficient document embedding, leveraging its compact yet powerful transformer architecture to generate high-quality sentence representations for topic modeling [37,38]. The BERTopic process consists of three steps: (1) document embedding, (2) document clustering, and (3) topic representation [39,40]. Document embedding, using the all-MiniLM-L6-v2, reads sentences in both directions, pretrains them in deep representations, and then generates word embeddings (ie, representations of valued vectors transformed from words to numbers depending on the surrounding contexts). Document clustering applies Uniform Manifold Approximation and Projection to reduce the dimensions [41] and cluster semantically similar words through the Hierarchical Density-Based Spatial Clustering of Applications with the Noise algorithm [42]. Topic representation extracts significant words on class-based term frequency-inverse document frequency, which is a metric to measure the relevance of words [43].

To validate the findings, LDA—a widely used topic modeling method—was also applied. Since no definitive method exists to determine the number of topics, the research team conducted multiple discussions to assess topics generated by the BERT-based approach. The final number of topics was determined by considering evaluation metrics, intertopic distance maps, data characteristics, and interpretability of the results, in comparison to LDA [25,44,45]. The evaluation metrics used to assess topic quality were topic coherence and topic diversity, both ranging from 0 to 1. Topic coherence measures how frequently words co-occur in a dataset, with higher values indicating more interpretable topics, while topic diversity assesses the uniqueness of words within topics, with higher values indicating more distinct topics [46]. The intertopic distance map visualizes topics as circles in a 2D space, where the size of each circle represents the number of studies assigned to that topic, and the proximity between circles indicates the degree of similarity between topics [47]. In this study, the BERT-generated model demonstrated superior evaluation metrics and a more balanced distribution of topics in the intertopic distance maps, with minimal overlap. In addition, the BERT approach yielded more data-related, distinct, and plausible results, outperforming LDA topic modeling in both structure and longitudinal topic trends. In line with the common practice in prior studies using BERTopic, Topic -1—a nonthematic cluster of documents with weak thematic alignment and often containing words commonly found across different topics [48]—was excluded from the interpretation of results. The proportion of nonthematic documents assigned to Topic -1 varied among the studies, ranging from approximately zero to 54% [25,36]. In this study, the analyses were performed twice, with one aimed at broadening the understanding of the literature related to health care services for people living with HIV and the other focused on a specific exploration of the health care services described in the literature.

### Ethical Considerations

The study was exempted from the approval process because it was a review and analysis of existing literature. No human participants were not directly involved, and no personal or private information was used.

## Results

### Data Description

**Figure 1.**
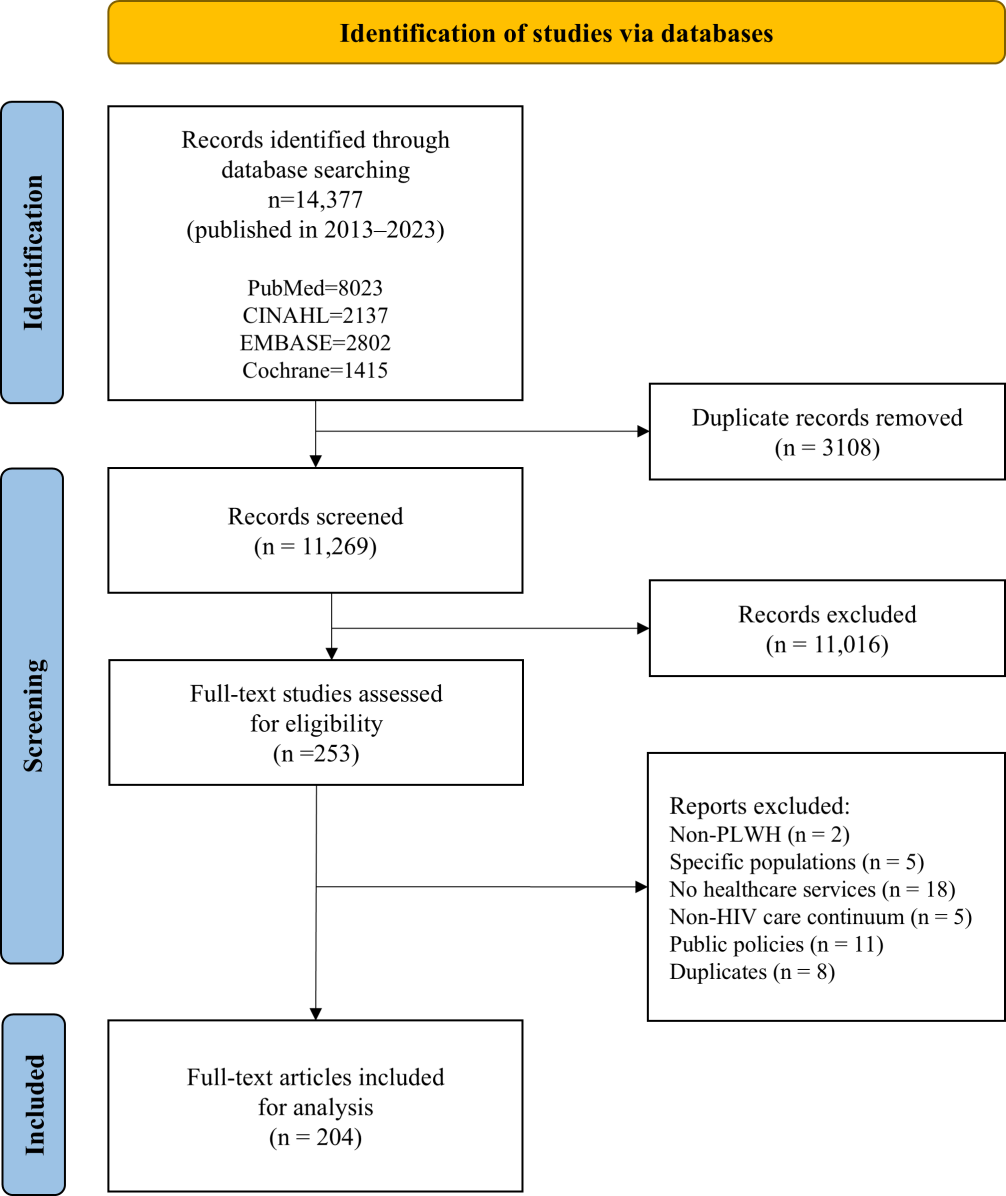
PRISMA flowchart for article selection.

**Figure 2.**
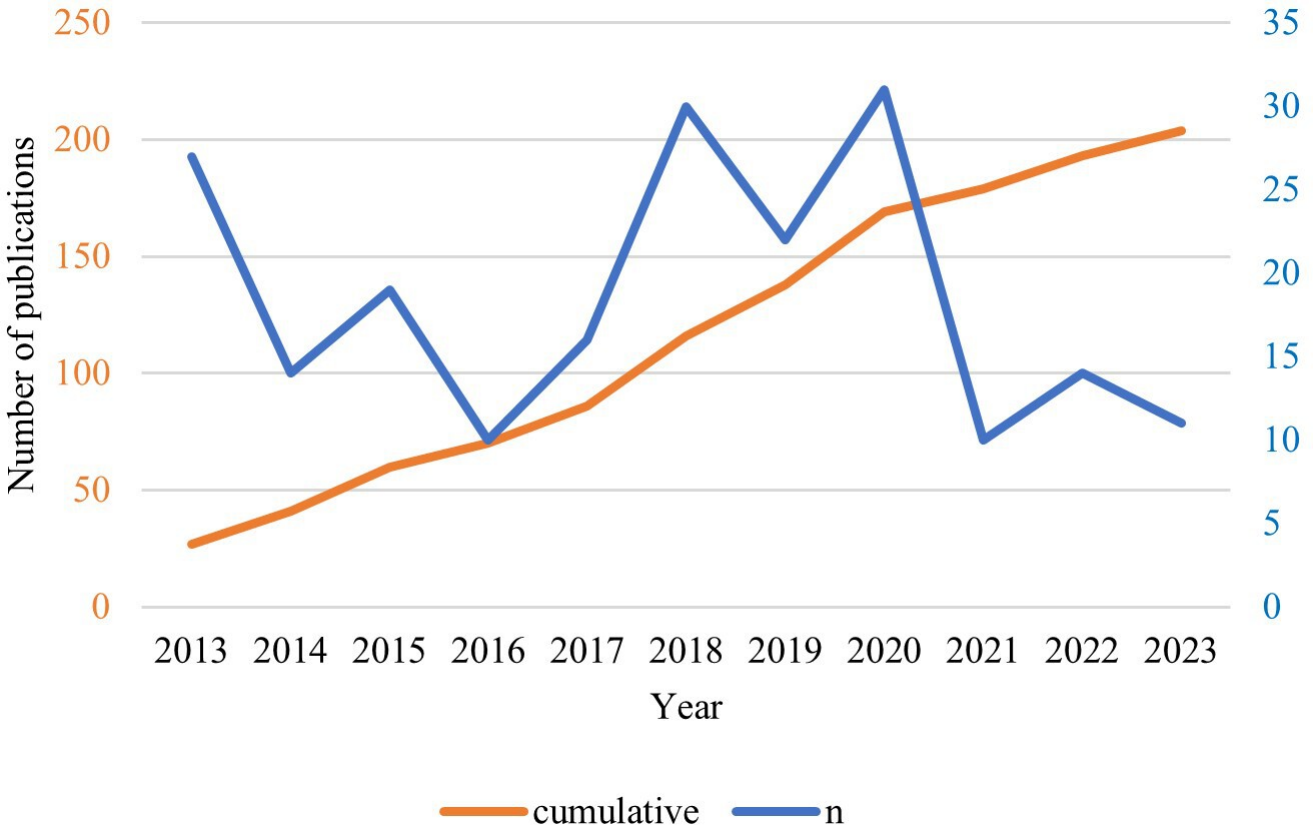
Number of publications on health care services for people living with HIV from 2013 to 2023.

A total of 204 studies were included in the analysis (Figure 1). Over the past decade, an average of approximately 19 studies on health care services for people living with HIV were conducted annually, ranging from 10 to 31 studies (Figure 2). The number of publications increased significantly between 2018 and 2020, but declined in 2016 and from 2021 onwards. Table 1 summarizes the characteristics of the included studies. The majority of studies were conducted in the United States (n=174, 85.3%). Regionally, as defined by the WHO [49], the Region of the Americas (AMR) accounted for the highest representation, while the Eastern Mediterranean Region was represented by only one study. The average age of study participants was 40.52 (SD 10.67) years, with the longest recorded duration since their HIV diagnosis being 30 years. Among the HIV care continuum stages, the treatment retention stage comprised the highest number of studies, totaling 185, while the long-term stage had the fewest studies.

**Table 1.** Characteristics of the analyzed studies (N=204).

Characteristics	Values
Research design, n (%)	
Quantitative	164 (80.4)
Qualitative	22 (10.8)
Mixed methods	18 (8.8)
Sample	
Size, min-max	10‐32,999
Age, years	18‐85
Period of HIV diagnosis, years	0‐30
Country, n (%)	
Region of the Americas, n (%)	
United States	174 (85.3)
Canada	6 (2.9)
European Region, n (%)	
Spain	8 (3.9)
United Kingdom	4 (2.0)
Denmark	1 (0.5)
Italy	1 (0.5)
Netherlands	1 (0.5)
Poland	1 (0.5)
Switzerland	1 (0.5)
Western Pacific Region, n (%)	
Australia	3 (1.5)
Korea	2 (1.0)
Singapore	1 (0.5)
Oman	1 (0.5)
HIV care continuum^a^, %	
Linkage to care	17
Treatment initiation	29
Treatment retention	185
Long-term	11

a Records duplicates.

### Topic Modeling on Health Care Services Research for People Living With HIV

BERTopic was initially applied to the abstract corpus to identify overarching trends and the knowledge structure within the included publications. Among the 242 documents, 191 were classified into 5 distinct topics, while the remaining 51 (21.1%) were identified as a nonthematic topic. This proportion is relatively small compared to previous studies, where the proportion ranged from 0% to 54% [25,36]. The most frequently occurring keywords within the nonthematic topic included “hiv,” “intervention,” “health,” “use,” and “improve,” which represent broad and general concepts rather than distinct thematic categories.

Table 2 presents the 5 distinct topics, labelled by the research team, alongside their respective keywords and the number of documents assigned to each topic. The 2 prominent topics—“ART adherence*”* and “Retention and engagement in care*”*—align with key stages of the HIV care continuum. The remaining 3 topics focus on specific populations: “Minorities-focused*”* consists of studies concentrating on race and ethnicities, while “Young population-focused,” emerged as an age-specific category. In addition, “Newly diagnosed men who have sex with men (MSM)*”* encompasses health care services tailored to a specific population at a specific stage.

**Table 2.** Topics and top keywords identified in health care services research for people living with HIV.

Topics	Top keywords (importance weights)	n
ART^a^ adherence	adherence (0.0470), intervention (0.0274), hiv (0.0267), art (0.0255), use (0.0229), medication (0.0221), group (0.0184), people living with HIV (0.0172), improve (0.0164), self (0.0158)	120 (62.8%)
Retention and engagement in care	retention (0.0575), hiv (0.0505), intervention (0.0344), clinic (0.0253), health (0.0226), peer (0.0221), program (0.0209), improve (0.0208), adult (0.0191), engagement (0.0175)	29 (15.2%)
Minorities-focused	hiv (0.0451), component (0.0357), intervention (0.0292), black (0.0251), interview (0.0241), american (0.0237), african (0.0219), african-american (0.0219), use (0.0214), latino (0.0209)	16 (8.4%)
Young population-focused	intervention (0.0641), adherence (0.0586), youth (0.0504), adolescent (0.0344), art (0.0342), hiv (0.0336), condition (0.0292), accept (0.0261), medication (0.0246), group (0.0240)	14 (7.3%)
Newly diagnosed MSM^b^	test (0.0572), hiv (0.0496), ltc (0.0428), clinic (0.0387), client (0.0341), msm (0.0303), diagnose (0.0301), hiv-test (0.0250), case (0.0234), men (0.0216)	12 (6.3%)

a ART: antiretroviral therapy.

b MSM: men who have sex with men.

When comparing the evaluation metrics of the models generated using LDA and BERTopic, the topic coherence scores were 0.2987 and 0.4264, respectively, while the topic diversity scores were 0.4200 and 0.6667, respectively, indicating superior performance of BERTopic in both aspects. In addition, the intertopic distance map of the BERT-generated model presented a more well-balanced distribution of topics with no overlap, whereas the LDA-generated model exhibited substantial topic overlap (Multimedia Appendix 1). Considering these comparisons, including topic interpretability, the BERTopic-generated model was determined to be the optimal choice for analysis.

**Figure 3.**
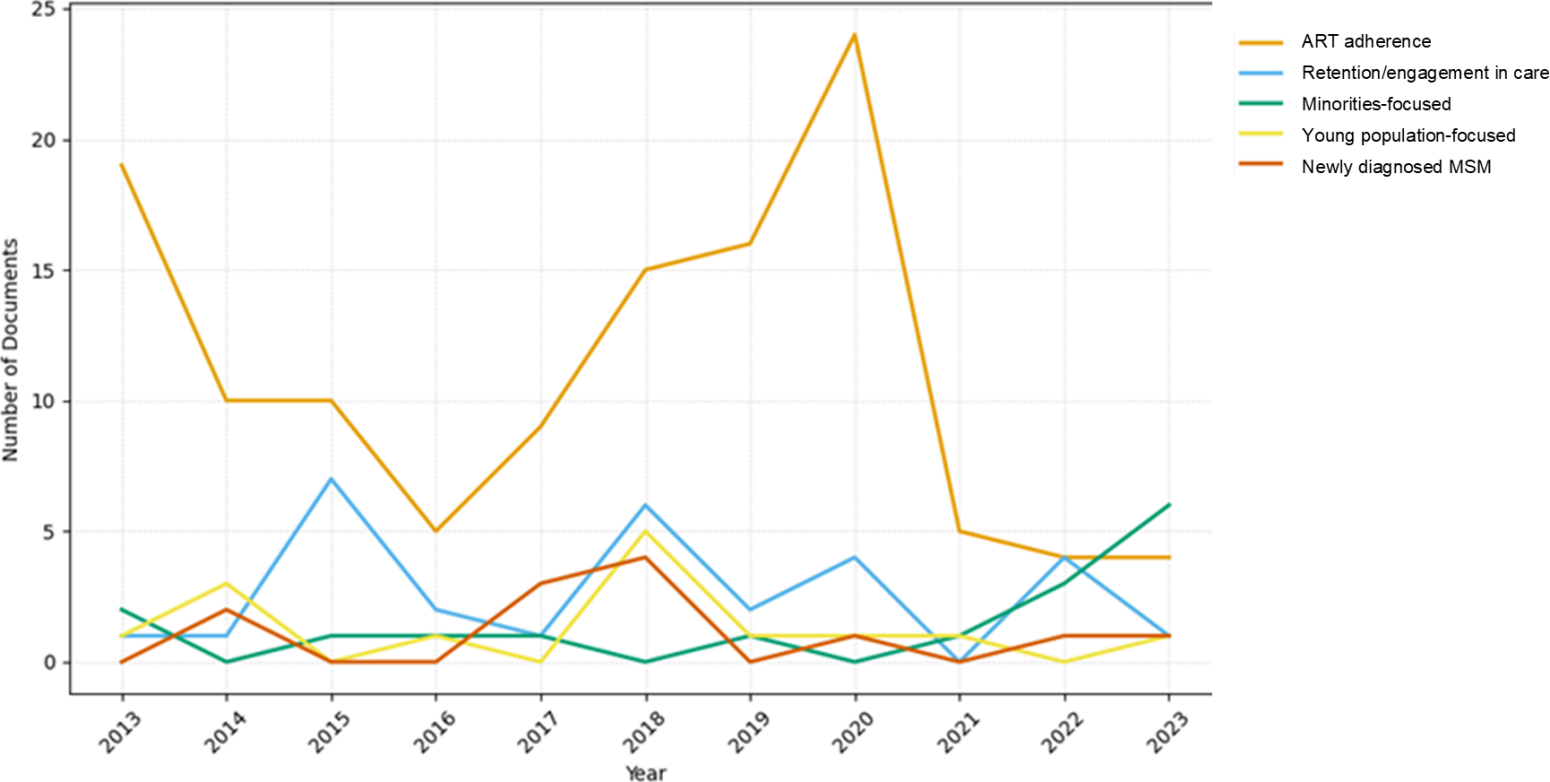
Topic distribution in health care services research for people living with HIV from 2013 to 2023. ART: antiretroviral therapy.

Figure 3 depicts the distribution of topics over the years. The research focus on “ART adherence*”* peaked in 2020, while notable declines were observed during mid-2010 and after 2020. “Retention and engagement in care” exhibited fluctuating trends. Coinciding with the onset of the COVID-19 pandemic, studies involving people living with HIV tended to decrease post 2020, but research on “Minorities-focused” topic exhibited recent increases.

**Figure 4.**
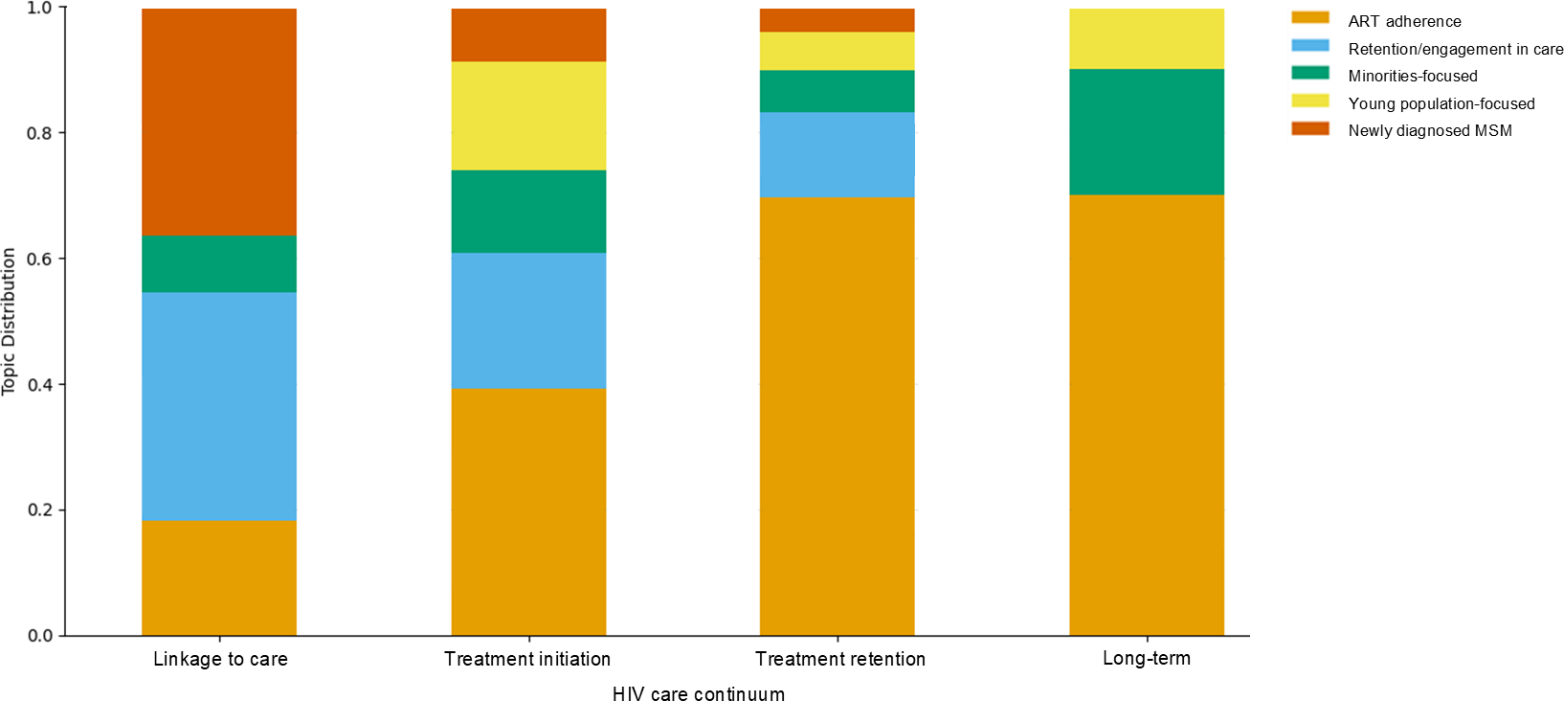
Distribution of topics in health care services research for people living with HIV across the HIV care continuum. ART: antiretroviral therapy; MSM: men who have sex with men.

Among the 4 stages of the HIV care continuum, the treatment initiation and retention stages were investigated across all topics (Figure 4). In the first stage (linkage to care), research on young populations was notably lacking, whereas the “Newly diagnosed MSM” topic had the highest representation compared to other stages. In the treatment initiation stage, the “Young population-focused” topic began to emerge, with all topics being more evenly distributed. The “ART adherence” topic accounted for more than half of the studies in the treatment retention stage. Finally, the long-term stage had the fewest studies overall. Regarding the regional distribution of topics by country, only the AMR accounted for all topics, while studies from other regions were limited. Research conducted in the AMR predominantly focused on ART adherence.

### Topic Modeling on Health Care Services for People Living With HIV

The second analysis aimed to provide more comprehensive examination of health care services by using the detailed descriptions of health care services found in the methods section of the articles. When running BERTopic, 181 out of the total 242 documents were categorized into 7 topics, while the remaining 61 (25.2%) fell into a nonthematic category. The most frequent occurring keywords in the nonthematic topic included “care,” “participant,” “hiv,” “patient,” and “health,” suggesting that these documents contained broad, general concepts rather than distinct thematic distinctions.

Table 3 presents the 7 topics, as labeled by the research team, along with their respective keywords and the number of documents assigned to each. Among these, “ART adherence and counseling*”* was the most prevalent topic, reflecting strategies to enhance ART adherence, as indicated by the keywords “counseling” and “message.” Other topics explore more specific aspects of health care service delivery models, target populations, and service content. The “Peer support” and “Pharmacist involvement” topics highlight the engagement of both health care and non-health care professionals in studies providing health care services to people living with HIV. The “Pharmacist involvement” and “Youth-to-adults transition care” topics specify who delivers health care services and for whom, respectively. “Monetary incentives” pertain to studies using financial rewards as benefits for people living with HIV. The topic “Health promotion and care navigation” focuses on structured interventions designed to guide individuals in maintaining and improving health. Finally, the “Linkage to care” represents studies addressing a specific stage of the HIV care continuum. As reflected in their labels, the 7 topics collectively reflect a combination of delivery methods and content in the provision of health care services to people living with HIV.

**Table 3.** Topics and top keywords identified as health care services for people living with HIV.

Topics	Top keywords (importance weights)	n
ART^a^ adherence and counseling	adherence (0.0312), participant (0.0283), session (0.0280), medication (0.0212), intervention (0.0209), use (0.0186), patient (0.0184), message (0.0150), counsel (0.0139), receive (0.0128)	81 (44.8%)
Peer support	care (0.0369), hiv (0.0344), peer (0.0246), health (0.0229), intervention (0.0211), support (0.0196), participant (0.0190), patient (0.0182), session (0.0176), barrier (0.0172)	40 (22.1%)
Youth-to-adults transition care	care (0.0572), hiv (0.0473), patient (0.0435), team (0.0329), clinic (0.0276), transition (0.0253), visit (0.0222), service (0.0213), provide (0.0200), adult (0.0183)	20 (11.0%)
Pharmacist involvement	art (0.0606), patient (0.0568), medication (0.0389), pharmacist (0.0382), hiv (0.0375), clinic (0.0304), day (0.0292), drug (0.0283), pharmd (0.0246), hiv-pharmd (0.0246)	17 (9.4%)
Monetary incentives	chance (0.0482), participant (0.0449), point (0.0440), chance-win (0.0437), win (0.0437), hiv (0.0416), component (0.0395), prize (0.0351), tm (0.0328), test (0.0320)	9 (5.0%)
Health promotion and care navigation	hiv-care (0.0662), care (0.0613), hiv (0.0611), health (0.0514), health-promotion (0.0493), promotion (0.0490), care-navigation (0.0480), message (0.0479), navigation (0.0435), participant (0.0434)	8 (4.4%)
Linkage to care	care (0.0684), hiv (0.0554), site (0.0545), people living with HIV (0.0407), hiv-care (0.0347), classify (0.0341), ltc (0.0336), ed (0.0312), program (0.0311), patient (0.0289)	6 (3.3%)

a ART: antiretroviral therapy.

When comparing the evaluation metrics of the models generated using LDA and BERTopic, the topic coherence scores were 0.3799 and 0.4458, respectively, while the topic diversity scores were 0.4429 and 0.6125, respectively, demonstrating BERTopic’s superior performance in both measures. Furthermore, the intertopic distance map of the BERT-generated model exhibited a well-balanced distribution of topics with no overlap, whereas the LDA-generated model displayed overlapping topics (Supplementary Figure 1 in Multimedia Appendix 1). Considering these factors, including topic interpretability, the BERTopic-generated model outperformed the LDA-generated model and was ultimately selected as the preferred approach.

**Figure 5.**
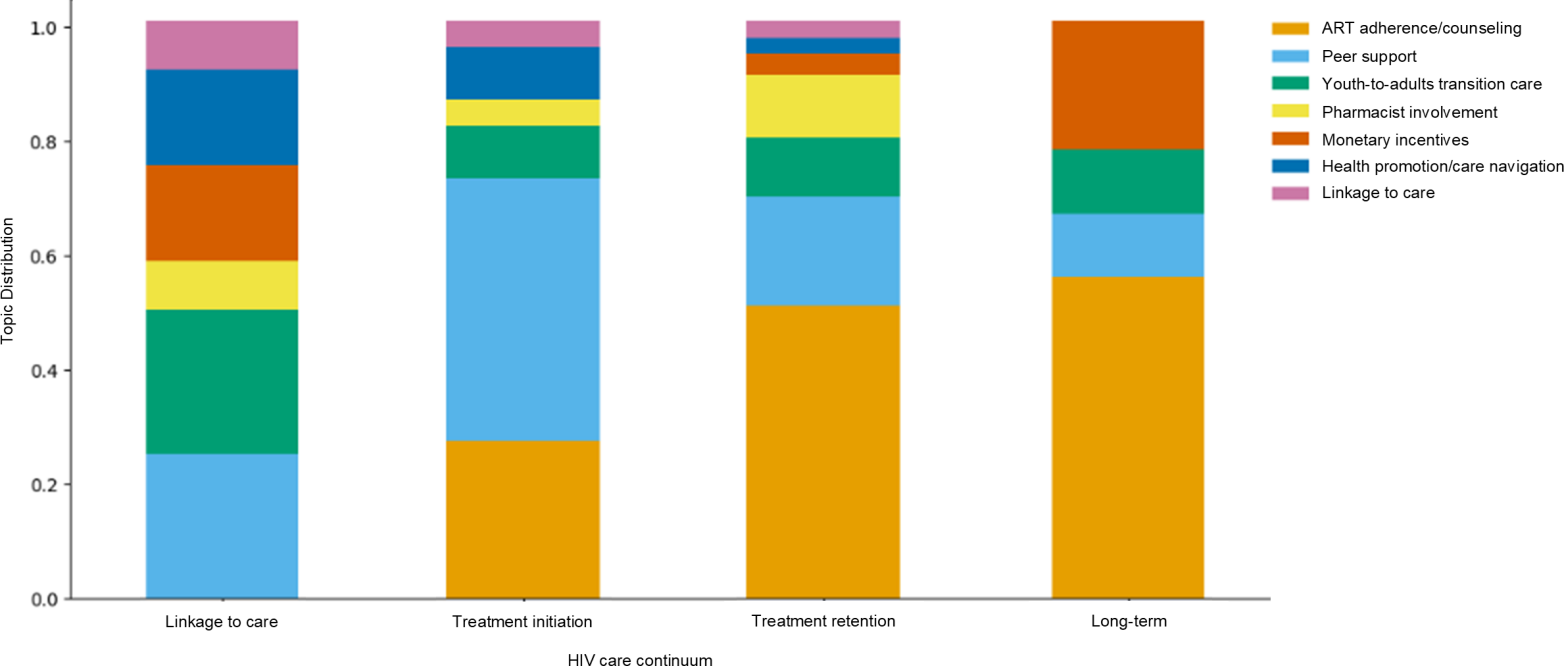
Distribution of health care service topics for people living with HIV across the HIV care continuum. ART: antiretroviral therapy; MSM: men who have sex with men.

Figure 5 illustrates the distribution of health care service topics across each stage of the HIV care continuum, highlighting varying levels of research coverage. While research on the initial linkage to care and final long-term stages were similarly scarce, the distribution of health care service topics presented distinct patterns. At the linkage to care stage, all health care services were represented except those related to medication adherence. In the treatment initiation stage, services focusing on medication adherence began to emerge, with peer support being the most prominent. During the treatment retention stage, medication adherence-focused services accounted for more than half of the studies. In the long-term stage, services related to medication adherence and monetary incentives became more prominent, whereas services involving pharmacist involvement and health navigation, which were present in earlier stages, declined. Overall, the diversity of health care services was the highest in the earlier stages of the continuum but became less varied in later stages, where research was more limited.

## Discussion

### Principal Findings

The HIV care continuum is a framework that describes the treatment journey of people living with HIV, encompassing the stages from diagnosis to linkage to care, engagement in care, and viral suppression through ART [7]. Key health care service components within this continuum include HIV testing, medication supply, treatment adherence monitoring, adverse event management, as well as virological monitoring [14-16]. However, like other chronic diseases, the HIV care continuum requires a shift toward an integrated care system centered on patient self-management, equipping individuals with the health care resources needed to manage their condition throughout life [50]. Moreover, as patients are increasingly expected to take active roles in managing their success of HIV treatment and long-term health [51], it is critical to move beyond the sole objective of viral suppression and emphasize overall quality of care for all people living with HIV [12]. Nonetheless, the extent to which current services align with the principles of lifelong continuity of care remains unclear. To address this gap, this study aimed to enhance the understanding of health care service trends and knowledge structures within the HIV care continuum by reviewing the literature using BERT. Given that approximately a decade has passed since the global recognition of the HIV care continuum, reviewing research in this field is essential for identifying both dominant areas and those lacking attention and support.

In this study, research on health care services within the HIV care continuum has primarily focused on the treatment retention stage, with a notable emphasis on ART adherence. While the importance of the HIV care continuum is widely recognized [12], research beyond disease-focused models remains underexplored in the field. Notably, despite longstanding concerns regarding the quality of life (QoL) of people living with HIV—including issues such as comorbidities, stigma, depression, discrimination, and sexual health [52]—these topics were not prominently represented in the extracted themes. The dominance of treatment-focused topics reveals persistent gaps in the literature, even amid global calls for more comprehensive, continuum-based HIV care. To broaden perspectives on life beyond treatment, future research should embrace emerging concepts such as “long-term success” [53] and “healthy living” [54] for people living with HIV, which emphasize a holistic approach addressing the multifaceted needs of people living with HIV. Optimizing health-related QoL and addressing age-related concerns is crucial to ensuring people living with HIV can lead fulfilling lives. This highlights the importance of multidisciplinary teams in delivering integrated, person-centered care and support for people living with HIV.

From a population perspective, there remains a notable lack of research on health care services tailored to minority populations. The topic of MSM emerged as a distinct research focus, reflecting the longstanding dominance of this demographic among people living with HIV. Consequently, research targeting MSM has been abundant. However, research addressing racial and ethnic minorities was consolidated into a single topic, and no distinct topic related to older adults emerged. This gap may be attributable to challenges in engaging these populations, particularly during the COVID-19 pandemic. Such academic trends inadequately address the global HIV epidemic. In high-income countries such as the US, HIV prevalence and incidence estimates are disproportionately higher among Black or African American and Hispanic or Latino individuals compared to White individuals. In addition, women are disproportionately affected among acquiring HIV through heterosexual contact, highlighting their vulnerability despite lower numerical representation [55]. The disease burden attributable to HIV infection among older adults, including prevalence, mortality, and QoL concerns, has increased [56]. These data underscore the critical need for comprehensive actions across the HIV care continuum and increased support tailored to younger and older populations, irrespective of race, ethnicity, and gender. Prioritizing and funding research aimed at understanding the specific needs of diverse and vulnerable populations is critical to fostering a more equitable health care landscape for HIV-infected individuals.

The topics identified on health care services aligned well with the needs of people living with HIV. Peer support and youth-to-adult transition services were prominent in the early stages of the HIV care continuum, when people living with HIV struggle with limited information following diagnosis and must decide whether to undergo ART. This period also represents a high-risk time for disengagement, particularly among young adults with HIV transitioning from pediatric to adult-oriented care [57,58]. ART adherence and counseling services were primarily implemented in the subsequent stages, supporting people living with HIV as they continued treatment and worked toward clinical stability [8]. While the health care services identified in the literature corresponded to different stages of the HIV care continuum, they were predominantly concentrated in the treatment retention stage, with fewer and less diverse services available in other stages. Therefore, more health care services should be developed and implemented across the HIV care continuum, particularly at the long-term stage. Specifically, more varied services to facilitate treatment initiation for people living with HIV at the initial stage are necessary, along with supportive services to promote healthy living and aging with HIV in the long term [59]. Furthermore, the cost-effectiveness, feasibility, sustainability, and confidentiality of these services must be further investigated, particularly for marginalized, younger, and older people living with HIV [60,61].

Pharmacists emerged as a distinct topic in health care services, whereas nurses and physicians were not separately identified. This may be attributed to the unique nature of HIV infection, which heavily relies on ART. Since the introduction of the first ART drug in 1987, ART has been continuously and actively developed and evaluated [62]. Furthermore, the WHO has continuously issued updated evidence-based ART guidelines since publishing its first consolidated guidelines in 2013 [33]. Recent trends have emphasized the cost-effectiveness and accessibility of ART, alongside advancements in potential cures and vaccines [62]. Given the dependency on ART and active trends in its advancement, pharmacies serve as crucial locations for the delivery of HIV treatment, testing, and prevention services, although the scope of services varies among countries. Pharmacists’ attitudes and services that are favorable to people living with HIV are essential for enhancing the HIV care continuum [63]. Expanding the services offered by pharmacies and pharmacists as primary service providers for testing and medication, particularly for high-risk and vulnerable populations, should be key consideration [64].

Although physicians and nurses play fundamental roles in HIV care, addressing evolving needs for health and longevity requires extending care beyond primary care to optimal long-term management beyond treatment. This need is particularly pertinent in high-income countries with abundant resources [65,66]. Nurses’ practices have been closely aligned with the needs of people living with HIV in HIV care, contributing to interdisciplinary efforts that facilitate the active development and implementation of interventions across the HIV care continuum [67]. However, their involvement in policymaking at local, national, and international levels remains limited [67]. Given their deep understanding of the needs of people living with HIV, nursing professionals should actively participate in decision-making processes to reshape practices and policies within the HIV care continuum. Furthermore, while not a distinct topic in this study, social welfare professionals also play a crucial role in the long-term management of HIV.

In addition, involving family members in health care services is recommended to optimize the HIV care continuum, particularly for key populations such as women and adolescents [10]. Numerous studies advocate a holistic patient-centered approach for people living with HIV, which involves not only pharmacists and peers, but also partners, family, and other individuals. Ensuring a comprehensive approach that encompasses a diverse range of health care and non-health care professionals over the long term is critical for promoting the HIV care continuum, particularly for vulnerable populations.

Overall, a decline in the number of studies on HIV services was observed, likely influenced by the impact of the COVID-19 pandemic. Notable drops in research output occurred between 2014 and 2016, and again in 2021, coinciding with global outbreaks of infectious diseases such as Ebola, Zika, and COVID-19 [68]. Similar to the upwards trend observed after the resolution of outbreaks such as Ebola and Zika, research efforts may increase shortly after the end of the COVID-19 pandemic. Therefore, research published from this perspective is important. As the vast majority of HIV research is conducted in the US, findings from this context may not be generalizable to other countries owing to significant sociocultural differences. Expanding research efforts across diverse countries and tailoring health care services to the specific needs of each country is crucial.

This study has several limitations. First, it focused solely on studies from high-income countries; studies from middle- and low-income countries may present different characteristics. Second, studies that did not explicitly address the HIV care continuum were excluded, necessitating careful interpretation of the findings. Finally, our trend analysis relied on linear models, potentially oversimplifying the intricate evolution of topics influenced by multiple factors.

### Conclusions

Despite the evolving nature of HIV care toward lifelong management, the literature predominantly emphasizes medical treatment, highlighting significant gaps in addressing the multifaceted needs of people living with HIV. This study provides a novel contribution by analyzing health care services aimed at enhancing HIV care continuum for people living with HIV using BERT, an advanced machine learning-based topic modeling method. By examining research trends across different stages of the HIV care continuum, this study offers valuable insights into both overarching research trends and specific service-related themes. Notably, it extends beyond traditional treatment-focused disease models to encompass long-term management and holistic well-being considerations, underscoring the importance of interdisciplinary collaboration in meeting the evolving needs of people living with HIV. Overall, this research advances understanding of HIV care and provides critical directions for future interventions and studies in this field.

## Supplementary material

10.2196/65081 Multimedia Appendix 1 Supplementary Figure 1. Comparison of inter-topic distance maps generated utilizing BERTopic and LDA.

10.2196/65081 Checklist 1 PRISMA-ScR (Preferred Reporting Items for Systematic reviews and Meta-Analyses extension for Scoping Reviews) Checklist.

## Abbreviations

AMR: Region of the Americas
ART: antiretroviral therapy
BERT: bidirectional encoder representations from transformers
LDA: latent dirichlet allocation
QoL: quality of life
TF-IDF: term frequency-inverse document frequency
WHO: World Health Organization
