# Supplementary figures and images for: Identifying Health Care Services Offered in the HIV Care Continuum via a Machine Learning–Based Topic Modeling Approach: Exploratory Literature Review
Source: JMIR Public Health Surveill. 2025 Jul 9;11:e65081. doi: 10.2196/65081 (PMC12266304; doi:10.2196/65081)

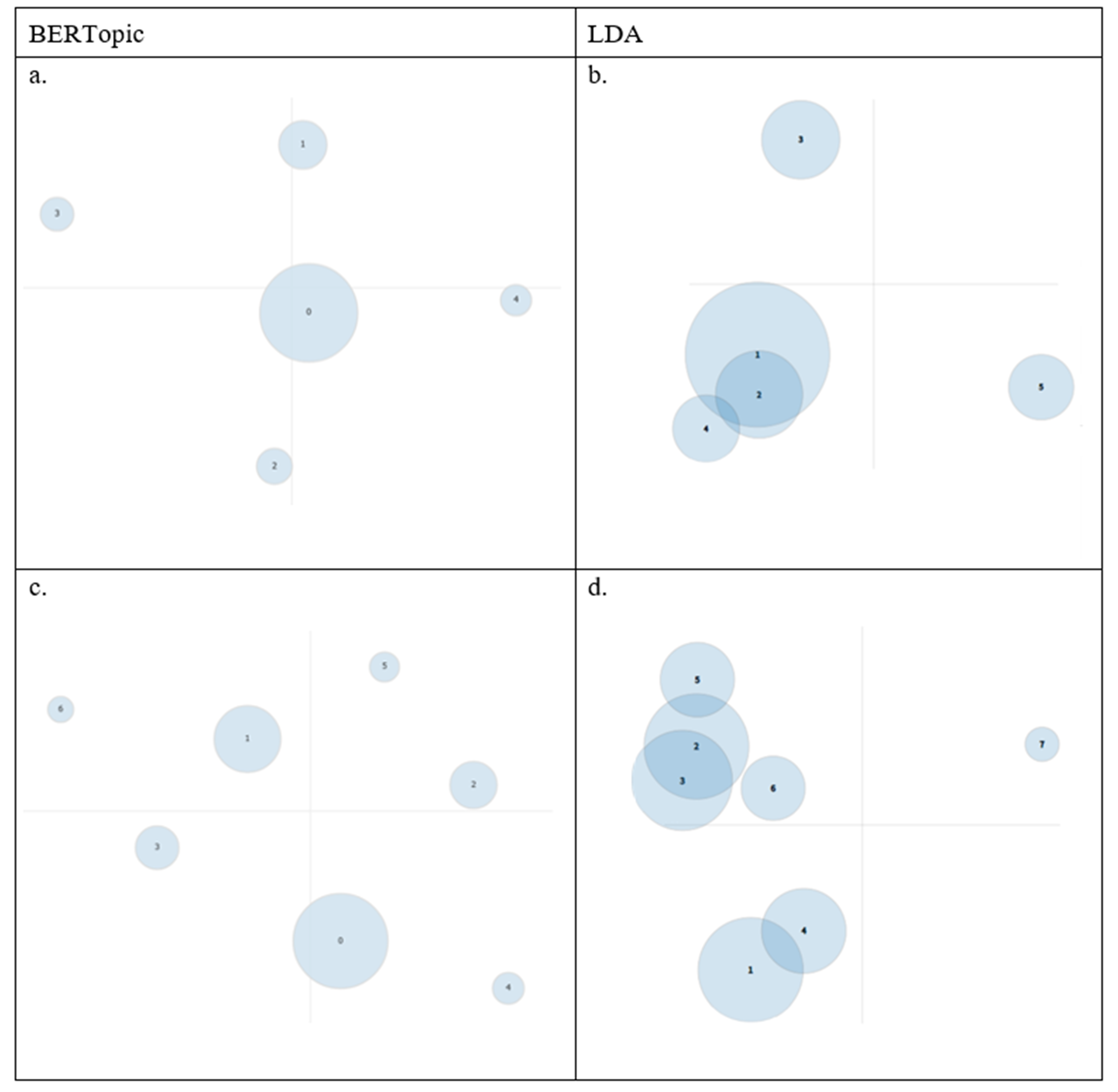

Supplement: Multimedia Appendix 1 [file publichealth-v11-e65081-s001.png]
